# Supplementary material for: Human cytomegalovirus in breast milk is associated with milk composition and the infant gut microbiome and growth
Source: Nat Commun. 2024 Jul 23;15:6216. doi: 10.1038/s41467-024-50282-4 (PMC11266569; doi:10.1038/s41467-024-50282-4)
Supplement: Supplementary file 1 — Supplementary Information [file 41467_2024_50282_MOESM1_ESM.pdf]

Supplementary material for:

Human Cytomegalovirus in breast milk is associated with milk composition and the infant gut microbiome and growth

K.E. Johnson, et al., *Nature Communications* 2024

Table of contents:

Supplementary Figure 1........ 2
Supplementary Figure 2........ 3
Supplementary Figure 3........ 4
Supplementary Figure 4........ 5
Supplementary Figure 5........ 6
Supplementary Figure 6........ 7
Supplementary Figure 7........ 8
Supplementary Figure 8........ 9
Supplementary Figure 9........ 10
Supplementary Figure 10........11
Supplementary Figure 11........12
Supplementary Figure 12........ 13
Supplementary Figure 13........ 14
Supplementary Figure 14........ 15
Supplementary Figure 15........ 16

Supplementary Figure 1.

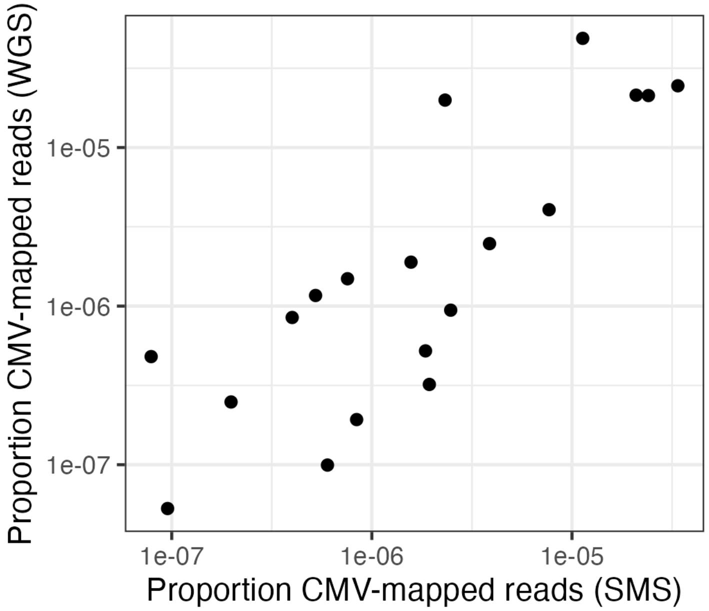

Supplementary Figure 1. Correlation between the proportion of CMV-mapped reads for CMV+ milk samples with sequencing data from both sources used in this study (N=24, Spearman's rho=0.81, P=3.47×10^{-5}).

Supplementary Figure 2.

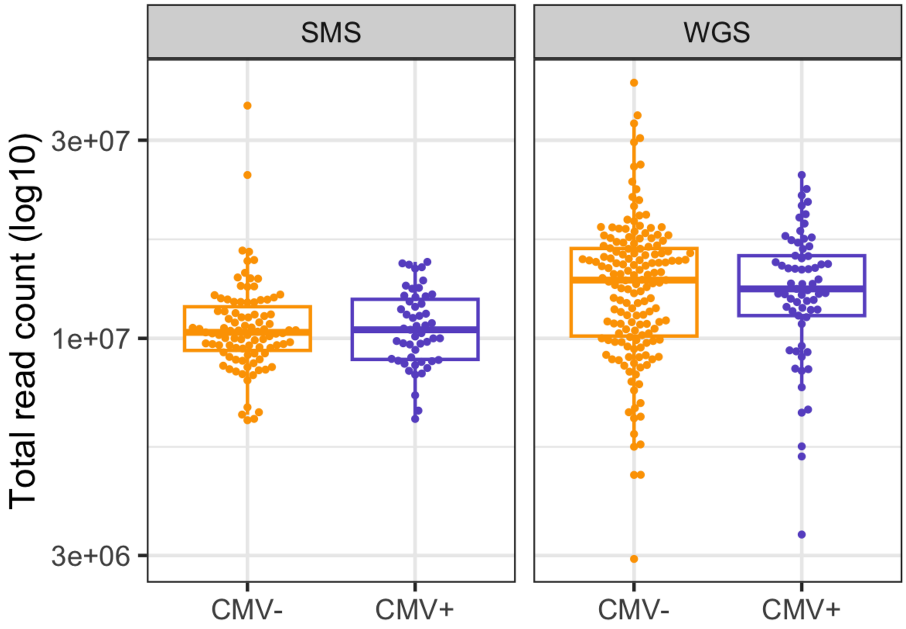

Supplementary Figure 2. The distribution of total read counts for CMV+ vs. CMV- milk samples from either shotgun metagenomic sequencing (SMS) or whole genome sequencing (WGS). There was no significant difference in mean total read count between CMV- and CMV+ milk samples in either dataset. SMS: CMV+ N=51, CMV- N=95; WGS: CMV+ N=65, CMV- N=159. There was no significant difference in read depth between CMV- and CMV+ samples using SMS (two-sided t-test; t=0.14; 95% C.I. -0.03, 0.04; p=0.89) nor WGS (two-sided t-test; t=0.34; 95% C.I. -0.04, 0.06; p=0.74). In boxplots, the thick center line represents the median, the upper and lower hinges represent the 75th and 25th percentiles, and the whiskers extend to the largest/smallest value no further than 1.5 times the interquartile range from the hinge.

Supplementary Figure 3.

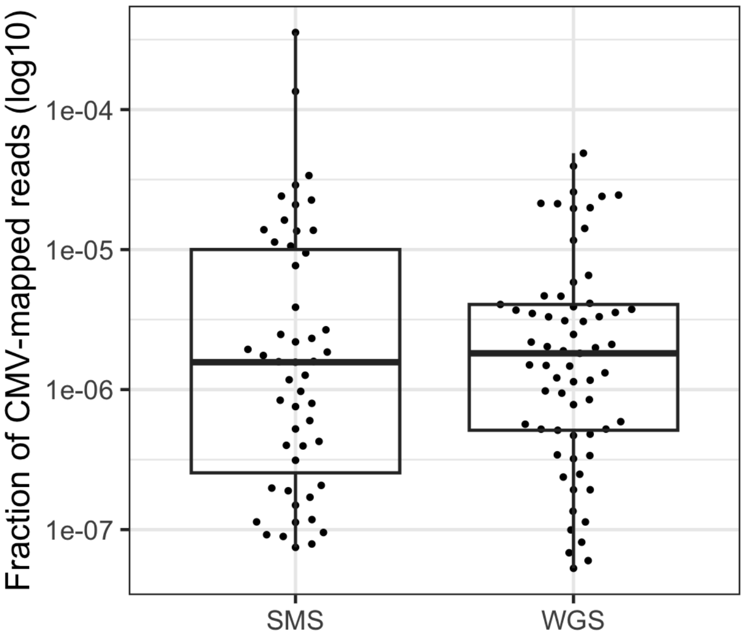

Supplementary Figure 3. The distributions of the proportion of CMV-mapped reads for milk samples with either shotgun metagenomic sequencing (SMS, N=51) or whole genome sequencing (WGS, N=65). There was no significant difference in the mean proportion of CMV-mapped reads between the two data types (two-sided t-test; t= -0.08; 95% C.I. -0.33, 0.30; p=0.93). In boxplots, the thick center line represents the median, the upper and lower hinges represent the 75th and 25th percentiles, and the whiskers extend to the largest/smallest value no further than 1.5 times the interquartile range from the hinge.

Supplementary Figure 4.

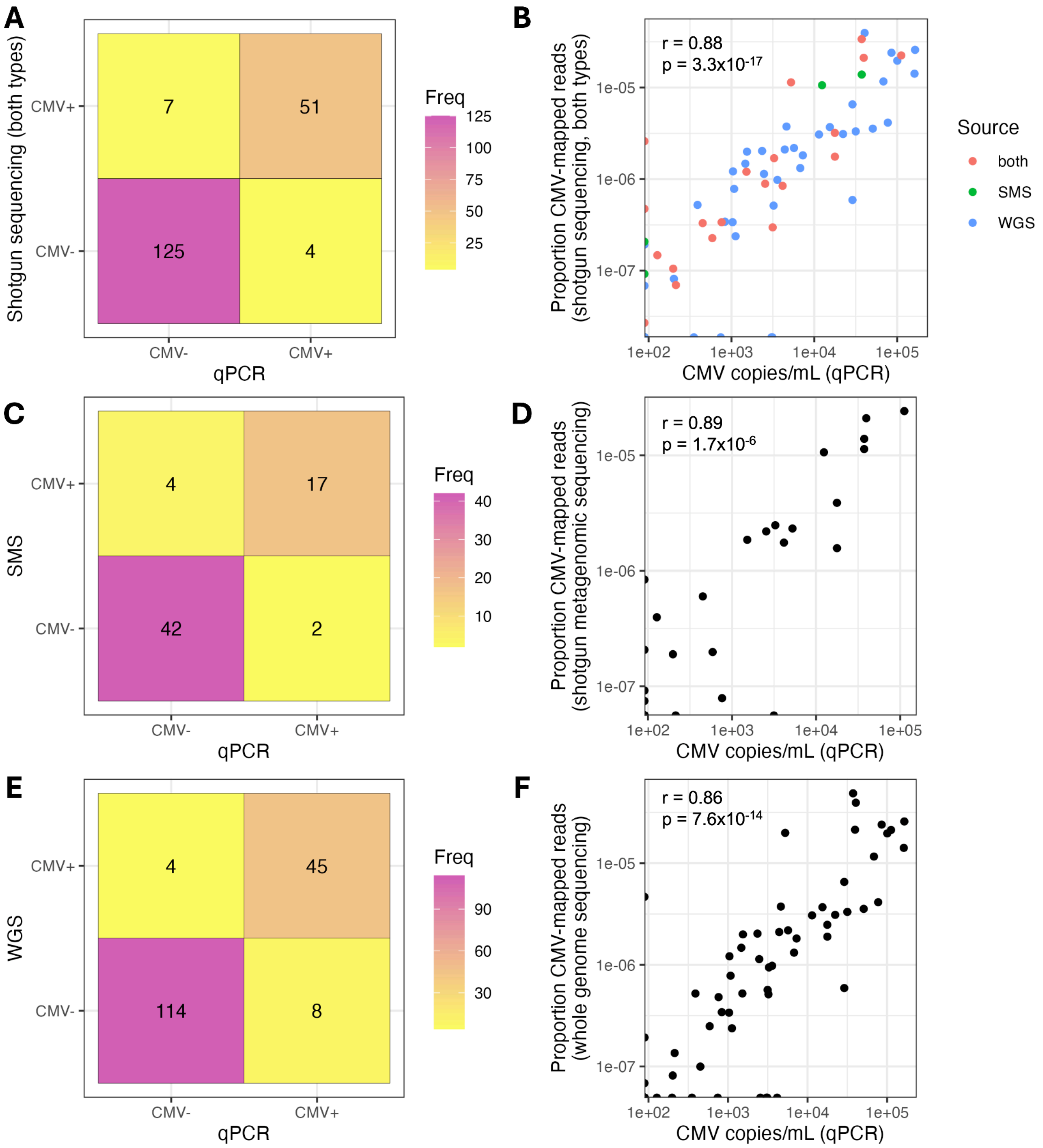

Supplementary Figure 4. Comparison of CMV detection in milk samples using shotgun sequencing data vs. qPCR. Left side column shows a confusion matrix of the count of samples detected as CMV+ vs. CMV- by each method. Right hand column shows the correlation between estimated viral load by qPCR vs. proportion of shotgun reads mapped to the CMV genome. Spearman correlation coefficients were calculated using only estimated viral load for samples that were CMV+ by both qPCR and shotgun sequencing, with two-sided p-values. Viral load estimates are plotted at log-10 scale on both axes. Each row represents a different shotgun data source (see Methods): **(A-B)** all shotgun data combined, N=187. In (B) samples are colored by the source of shotgun sequencing data. **(C-D)** only shotgun metagenomic sequencing data, SMS, N=65; **(E-F)** only whole genome sequencing data, WGS, N=171.

Supplementary Figure 5.

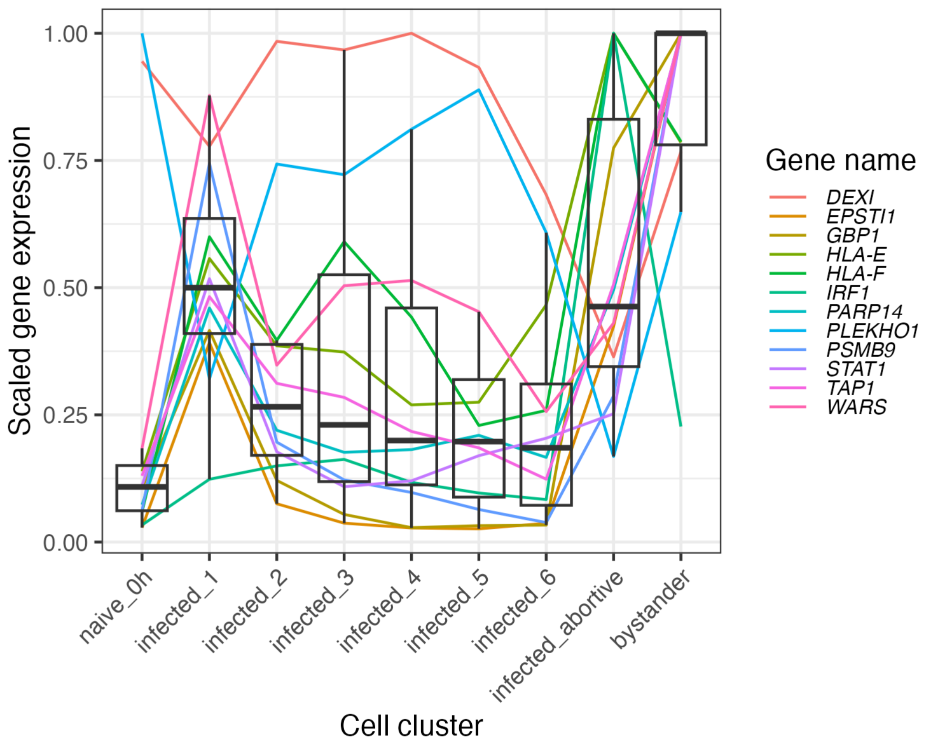

Supplementary Figure 5. For 12 genes that were upregulated in our CMV+ milk samples, this plot shows expression patterns across cell clusters in a publicly available dataset of single cells (human fibroblasts) exposed to CMV (Hein & Weissman, 2022). The x-axis groups are the 9 cell type clusters identified in the single cell dataset; naive_0h: cells before CMV infection; infected_1, …, infected_6: cell clusters along the CMV infection trajectory; infected_abortive: cells who are initially infected by CMV but the infection does not proceed to viral replication; bystander: uninfected cells that have high expression of interferon response genes due to signaling from nearby infected cells. The y-axis is expression values, scaled relative to the expression level in the cell cluster where each gene was most highly expressed. 10 out of 12 genes were most highly expressed in the 'bystander' cluster, which were defined as cells that did not have viral gene expression but did express high levels of interferon response genes. In boxplots, the thick center line represents the median, the upper and lower hinges represent the 75th and 25th percentiles, and the whiskers extend to the largest/smallest value no further than 1.5 times the interquartile range from the hinge.

Supplementary Figure 6.

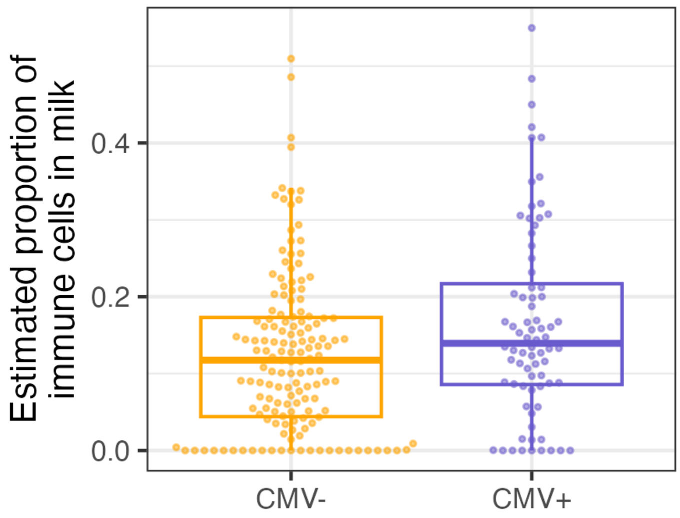

Supplementary Figure 6. The estimated proportion of immune cells in milk for CMV+ (N=76, purple) vs. CMV- (N=151, orange) milk samples. Cell type proportions were estimated via deconvolution of bulk RNA-sequencing data using a publicly available reference human milk single cell RNA-sequencing dataset (Nyquist et al., 2022). In boxplots, the thick center line represents the median, the upper and lower hinges represent the 75th and 25th percentiles, and the whiskers extend to the largest/smallest value no further than 1.5 times the interquartile range from the hinge.

Supplementary Figure 7.

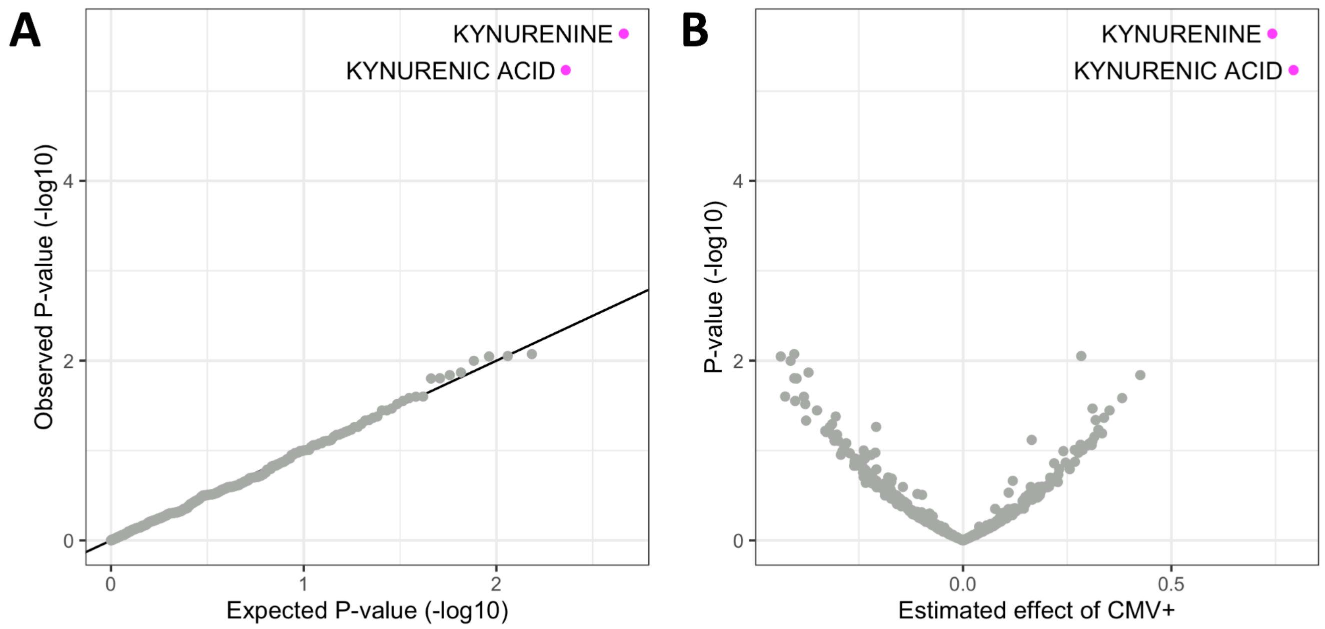

Supplementary Figure 7. (A) QQ-plot from the results of differential abundance analysis comparing metabolites in CMV+ and CMV- milk samples. The x-axis plots the expected P-value for the number of metabolites tested following a uniform distribution of P-values from 0 to 1, and the y-axis plots the observed P-values. **(B)** A volcano plot showing estimated effect sizes of CMV+ on milk metabolite abundance (x-axis) with each metabolite's P-value (y-axis). Metabolites whose P-value was below the false discovery rate threshold of 5% are colored in magenta.

Supplementary Figure 8.

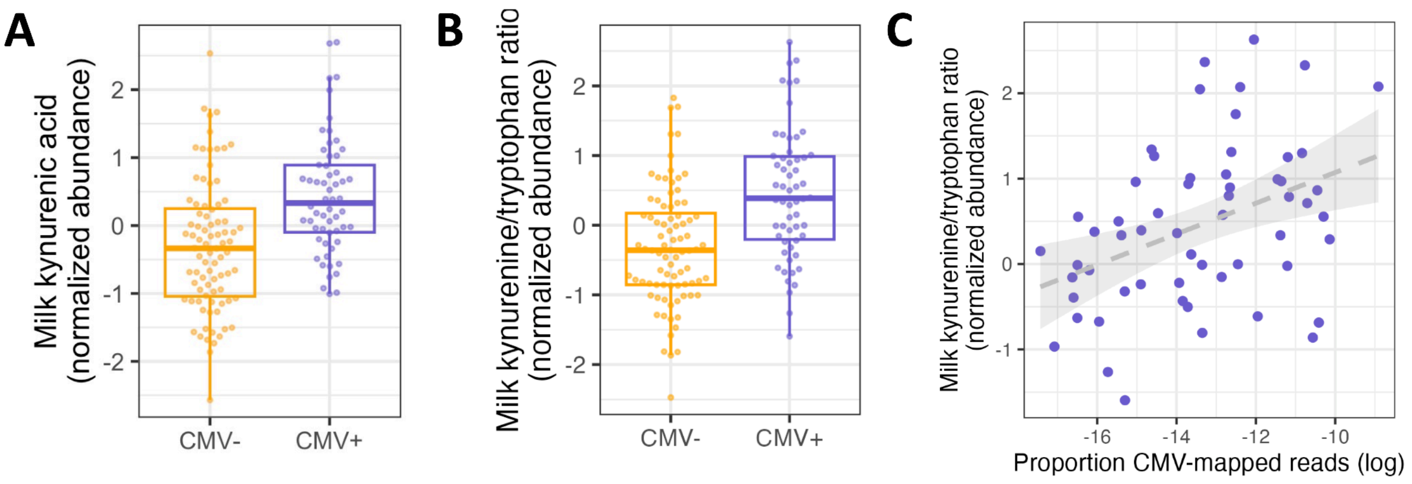

Supplementary Figure 8. (A) Kynurenic acid abundances in CMV- (N=84, orange) vs. CMV+ (N=58, purple) milk samples (beta = 0.75, P= 1.6×10^{-5}, q-value= 6.6×10^{-3}). Each dot represents a milk sample. **(B)** CMV+ milk samples (N=58, purple) had a higher ratio of kynurenine/trytophan abundances compared to CMV- (N=84, orange) (beta = 0.83, P = 2.7×10^{-6}). Each dot represents a milk sample. **(C)** We observed a positive correlation between the proportion of CMV-mapped reads in each CMV+ milk sample (x-axis) and the ratio of kynurenine/tryptophan abundances (y-axis) (N = 58, Beta = 0.19, P = 6.3×10^{-3}; two-sided p-value from linear regression). Each dot represents a milk sample, and only CMV+ milk samples (purple) were included in this analysis. The shaded gray area is the linear regression 95% confidence interval. All plotted metabolite abundances are residuals after correcting for covariates included in the association analysis with milk CMV status (see Methods). In boxplots, the thick center line represents the median, the upper and lower hinges represent the 75th and 25th percentiles, and the whiskers extend to the largest/smallest value no further than 1.5 times the interquartile range from the hinge.

Supplementary Figure 9.

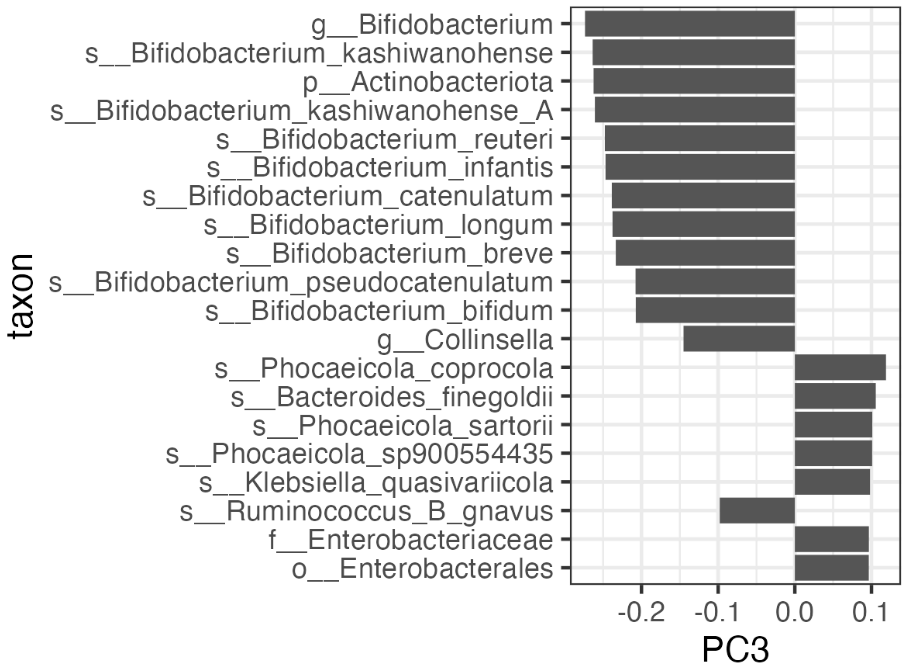

Supplementary Figure 9. The top 20 loading taxa for PC3 of the 1 month infant fecal metagenomes. For each taxon, the bar magnitude and direction represents its loading on PC3. Taxa are sorted in order of greatest (top) to smallest (bottom) magnitude, with the top 20 taxa included in this plot.

Supplementary Figure 10.

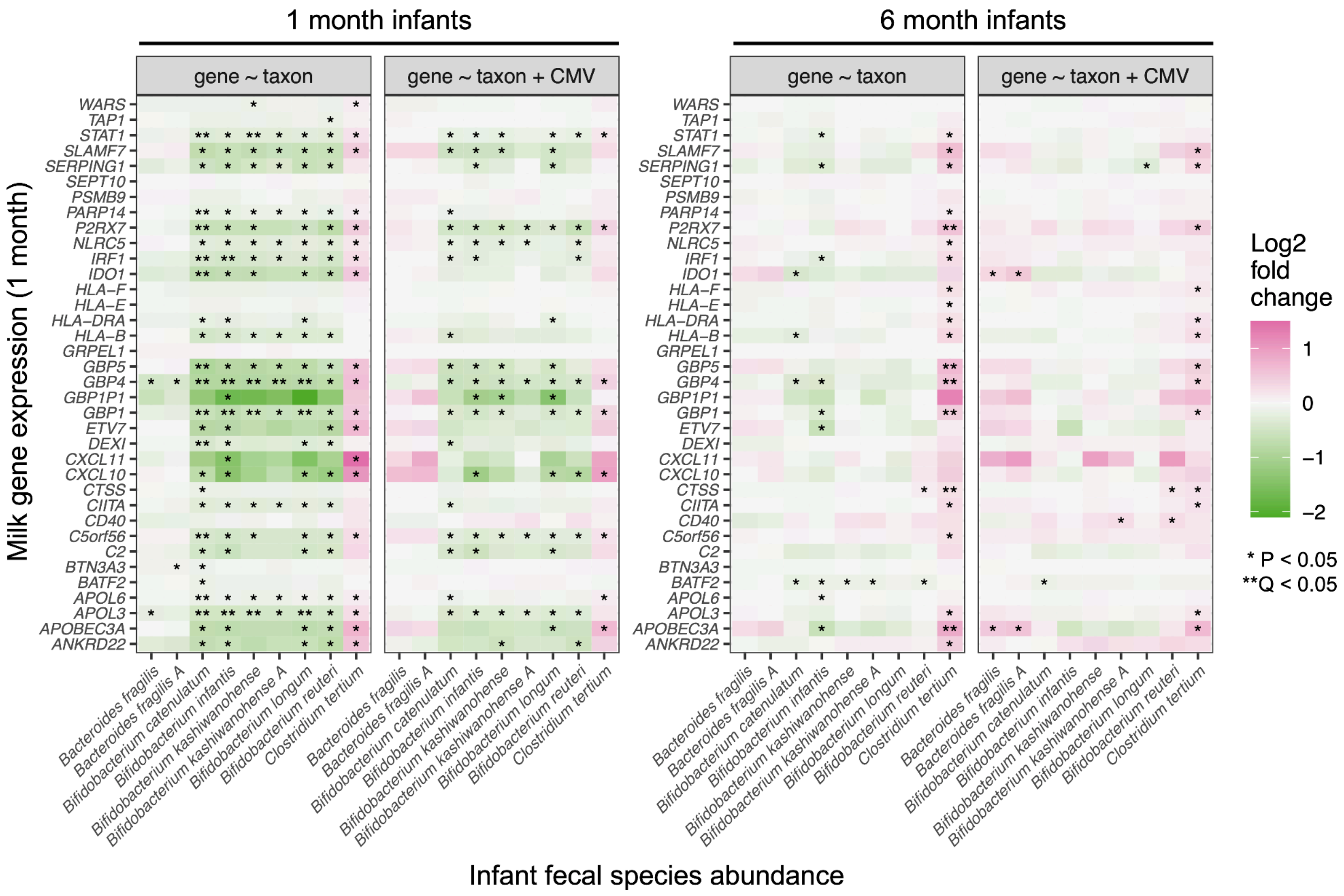

Supplementary Figure 10. Correlations between CMV-associated infant fecal microbial species (x-axis) and 36 CMV-associated milk-expressed genes (y-axis). Correlations were calculated for infant metagenomes at 1 (N=104) and 6 months (N=107), with ('gene ~ taxon + CMV') and without ('gene ~ taxon') milk CMV status as a covariate in DESeq2 (see Methods for all included covariates). Taxon names ending in 'A' were identified as distinct species by sequence identity in the reference genome database (see Methods).

Supplementary Figure 11.

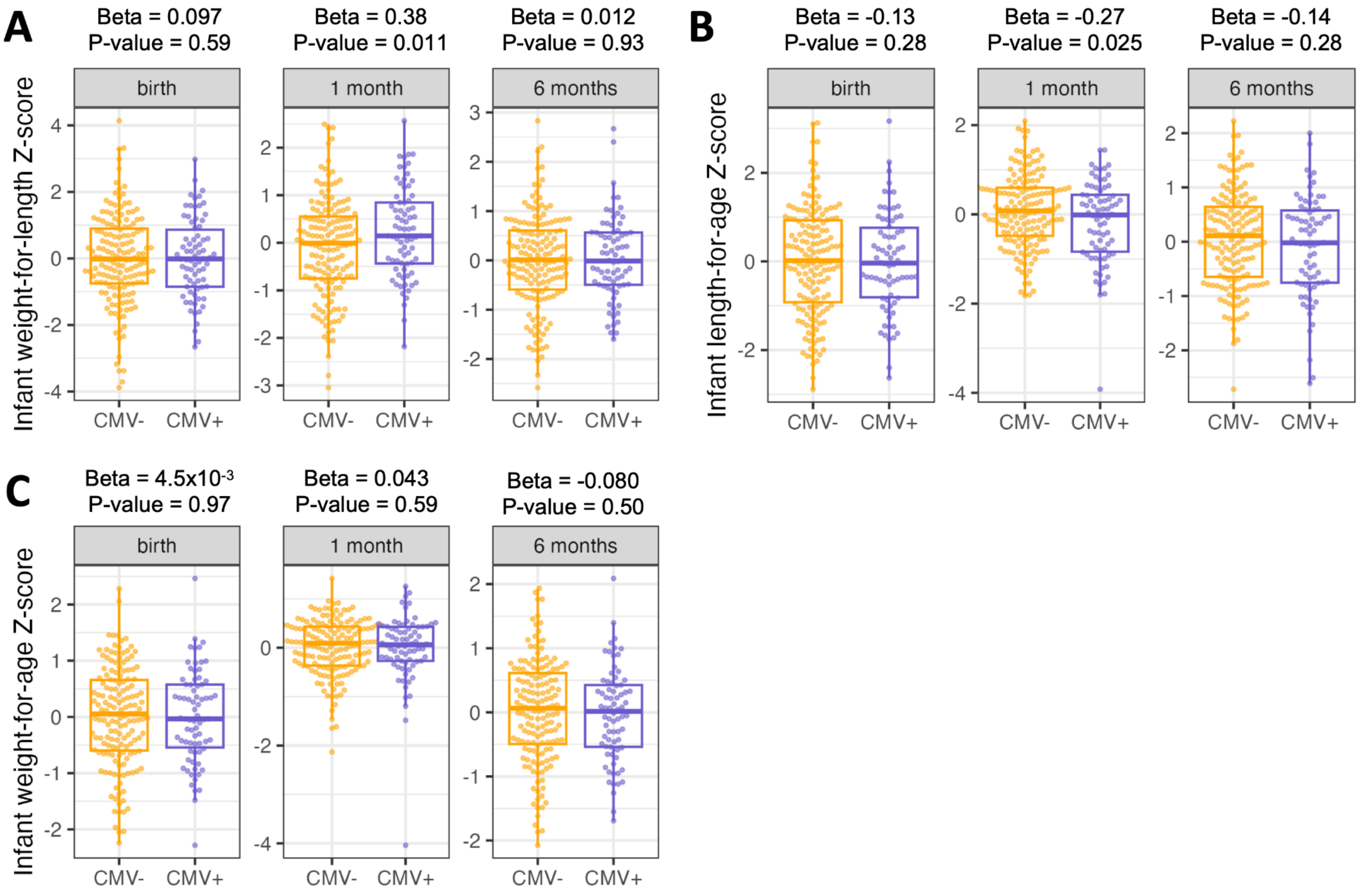

Supplementary Figure 11. Results of multivariate regressions of infant growth metrics at birth, 1 month of age, or 6 months of age vs. milk CMV status at 1 month postpartum. All regression models included the equivalent Z-score at birth as a covariate (except Z-score at birth). All plotted infant growth metrics are residuals after correcting for covariates included in the association analyses with milk CMV status (see Methods). In boxplots, the thick center line represents the median, the upper and lower hinges represent the 75th and 25th percentiles, and the whiskers extend to the largest/smallest value no further than 1.5 times the interquartile range from the hinge. Orange dots are CMV+ dyads (N=74), purple dots are CMV- dyads (N=155).

Supplementary Figure 12.

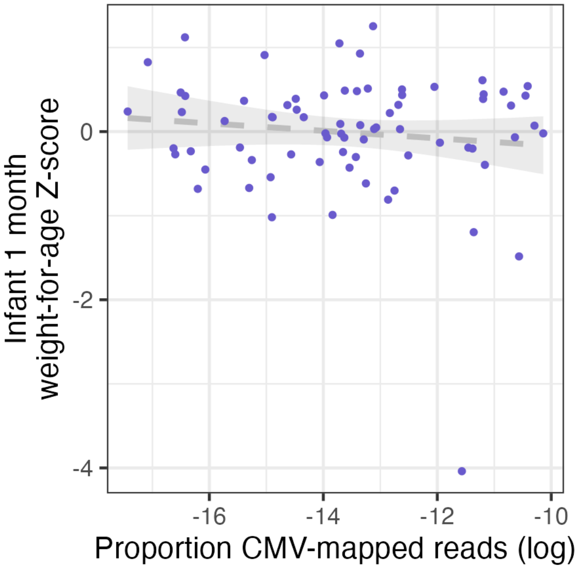

Supplementary Figure 12. Within infants fed CMV+ milk, there was no correlation between the proportion of CMV-mapped reads and infant weight-for-age Z-score at 1 month of age (N = 74, Beta = -0.035, P = 0.46). Plotted infant growth metrics are residuals after correcting for covariates included in the association analysis with milk CMV status (see Methods). Gray area indicates the 95% confidence interval.

Supplementary Figure 13.

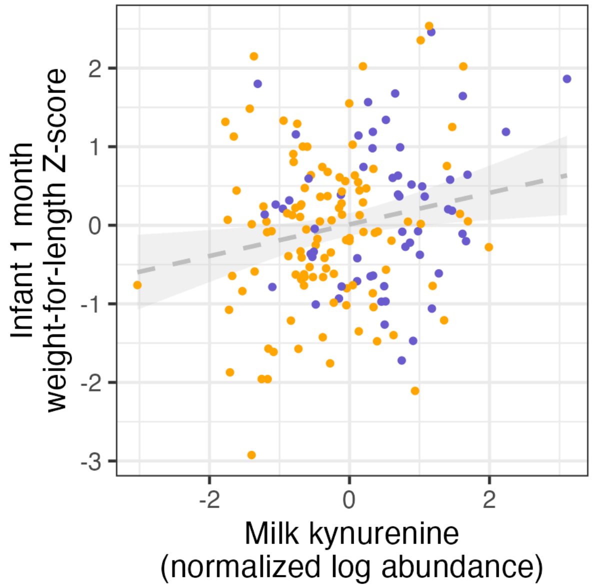

Supplementary Figure 13. There was a positive correlation between milk kynurenine (x-axis) and infant 1-month WLZ (y-axis). Each point represents a mother/infant pair, CMV- milk in orange (N=110), CMV+ milk in purple (N=58). Plotted infant growth metrics are residuals after correcting for covariates included in the association analysis with milk CMV status (see Methods). Gray area indicates the 95% confidence interval.

Supplementary Figure 14.

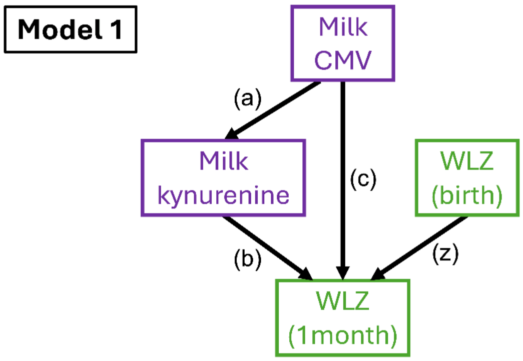

Parameter	Estimate	P-value		Model fit index	Value (P-value)
a	0.78	1.4×10^{-8}		AIC	1110.33
b	0.14	0.057		X^2 (1 d.f.)	0.10 (0.75)
c	8.6×10^{-3}	0.96		CFI	1.00
z	0.22	1.4×10^{-5}		NFI	0.99
a*b (mediated effect)	0.11	0.072		RMSEA	0.00
c+(a*b) (total effect)	0.12	0.41		SRMR	0.01

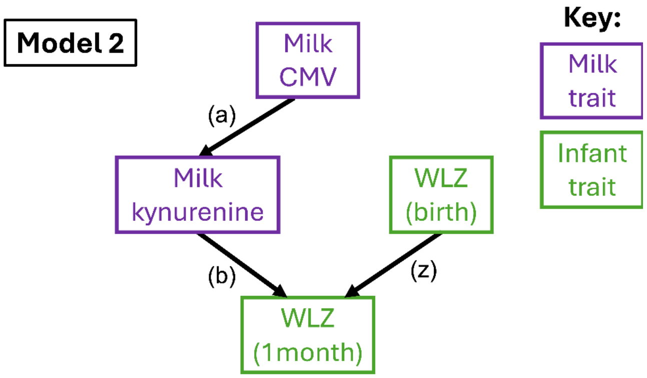

Parameter	Estimate	P-value		Model fit index	Value (P-value)
a	0.78	1.4×10^{-8}		AIC	1108.33
b	0.14	0.038		X^2 (2 d.f.)	0.10 (0.95)
				CFI	1.00
z	0.22	1.4×10^{-5}		NFI	0.99
a*b (mediated effect)	0.11	0.052		RMSEA	0.00
				SRMR	0.01

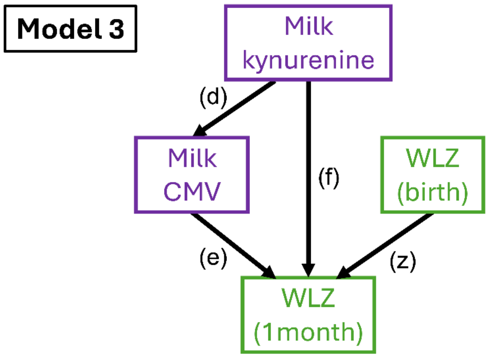

Parameter	Estimate	P-value		Model fit index	Value (P-value)
d	0.18	1.4×10^{-8}		AIC	817.215
e	8.6×10^{-3}	0.96		X^2 (1 d.f.)	0.02 (0.88)
f	0.14	0.057		CFI	1.00
z	0.22	1.4×10^{-5}		NFI	1.00
d*e (mediated effect)	1.5×10^{-3}	0.96		RMSEA	0.00
f+(d*e) (total effect)	0.14	0.038		SRMR	0.003

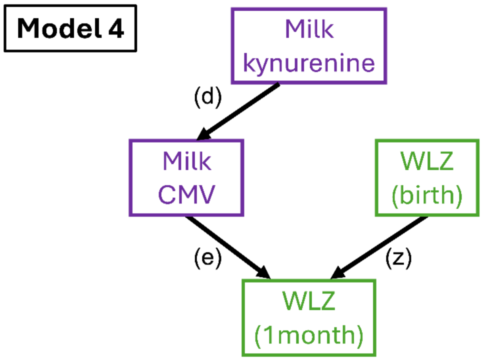

Parameter	Estimate	P-value		Model fit index	Value (P-value)
d	0.18	1.4×10^{-8}		AIC	818.793
e	0.12	0.41		X^2 (2 d.f.)	3.60 (0.17)
				CFI	0.97
z	0.22	1.3×10^{-5}		NFI	0.93
d*e (mediated effect)	0.021	0.42		RMSEA	0.063
				SRMR	0.037

Supplementary Figure 14. Structural equation modeling of milk CMV status, milk kynurenine abundance, and infant weight-for-length z-score (WLZ) at birth and 1 month of age. Models were evaluated using the R package 'lavaan' (see Methods) with a sample size of 200 mother-infant pairs. The best fit model (by lowest AIC, Model 3) is highlighted with the orange box. Model 3 found no significant relationship from milk CMV status to 1-month WLZ (parameter 'e'); and fits the data better than Models 1-2 that had kynurenine mediate any relationship between CMV and 1-month WLZ. Model 3 did have a significant total effect of kynurenine on 1-month WLZ (P = 0.038). Thus, we conclude that the association observed between CMV and 1-month WLZ when kynurenine was not included in the regression model (Figure 5A) is due to the difference in kynurenine levels between CMV+ and CMV- milk. AIC, Akaike information criterion; X^2, chi-squared test, CFI, comparative fix index; NFI, normed fit index; RSMEA, root-mean-square error of approximation; SRMR, standardized root-mean residuals. Purple boxes indicate milk traits, green boxes infant traits.

Supplementary Figure 15.

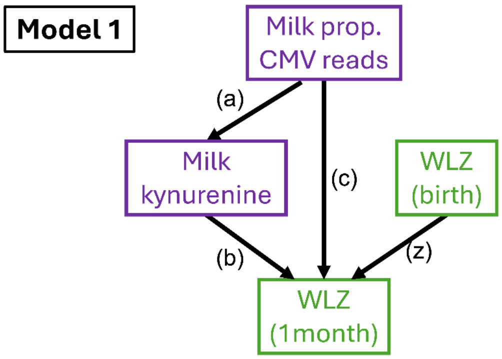

Parameter	Estimate	P-value	Model fit index	Value (P-value)
a	0.25	0.016	AIC	411.76
b	0.11	0.34	X^2 (1 d.f.)	0.27 (0.61)
c	-0.24	0.025	CFI	1.00
z	0.20	0.015	NFI	0.98
a*b (mediated effect)	0.027	0.38	RMSEA	0.00
c+(a*b) (total effect)	-0.22	0.040	SRMR	0.02

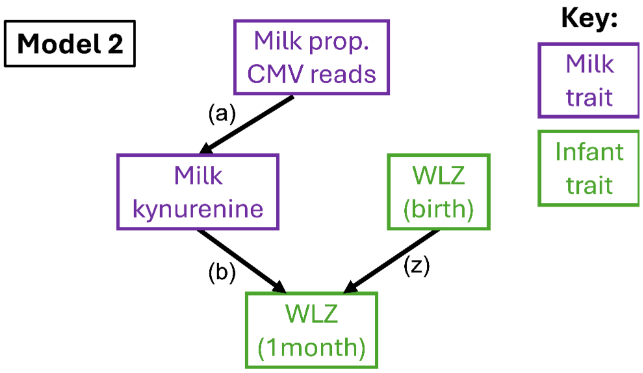

Parameter	Estimate	P-value	Model fit index	Value (P-value)
a	0.25	0.016	AIC	414.63
b	0.040	0.73	X^2 (2 d.f.)	5.14 (0.08)
			CFI	0.72
z	0.20	0.019	NFI	0.68
a*b (mediated effect)	0.010	0.73	RMSEA	0.14
			SRMR	0.08

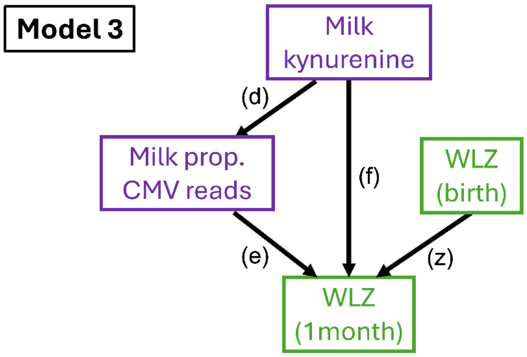

Parameter	Estimate	P-value	Model fit index	Value (P-value)
d	0.28	0.016	AIC	420.48
e	-0.24	0.025	X^2 (1 d.f.)	0.00 (0.96)
f	0.11	0.34	CFI	1.00
z	0.20	0.015	NFI	1.00
d*e (mediated effect)	-0.069	0.10	RMSEA	0.00
f+(d*e) (total effect)	0.041	0.72	SRMR	0.002

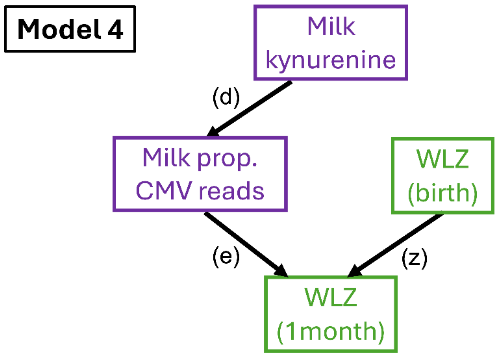

Parameter	Estimate	P-value	Model fit index	Value (P-value)
d	0.28	0.016	AIC	419.37
e	-0.22	0.040	X^2 (2 d.f.)	0.89 (0.64)
			CFI	1.00
z	0.19	0.018	NFI	0.94
d*e (mediated effect)	-0.061	0.18	RMSEA	0.00
			SRMR	0.03

Supplementary Figure 15. Structural equation modeling of the proportion of CMV-mapped reads in CMV+ milk samples (“Milk prop. CMV reads”, an estimate of viral load), milk kynurenine abundance, and infant weight-for-length z-score (WLZ) at birth and 1 month of age. Models were evaluated using the R package ‘lavaan’ (see Methods) with a sample size of 76 mother-infant pairs. The best performing model (by lowest AIC, Model 1) is highlighted with the orange box. Model 1 found evidence for both a mediating effect of kynurenine between milk CMV viral load and 1-month WLZ (‘a*b’), and a direct effect from viral load to 1-month WLZ (‘c’). AIC, Akaike information criterion; X^2, chi-squared test, CFI, comparative fix index; NFI, normed fit index; RSMEA, root-mean-square error of approximation; SRMR, standardized root-mean residuals. Purple boxes indicate milk traits, green boxes infant traits.
