# Supplementary material for: Human cytomegalovirus in breast milk is associated with milk composition and the infant gut microbiome and growth
Source: Nat Commun. 2024 Jul 23;15:6216. doi: 10.1038/s41467-024-50282-4 (PMC11266569; doi:10.1038/s41467-024-50282-4)
Supplement: Supplementary file 3 — Description of Additional Supplementary Files [file 41467_2024_50282_MOESM3_ESM.docx]

**Description of Supplementary Data**

***Filename:*** CMV_milk_genomics_suppData_1-13.xlsx

***Description:***

**Supplementary Data 1.** Milk sample CMV status data and other metadata. **indID**: mother/infant pair unique ID. **sms.cmv**: count of reads mapping to CMV genome from milk shotgun metagenomic sequencing data. **wgs.cmv**: count of reads mapping to CMV genome from milk whole genome sequencing data. **sms.tot**: total reads in milk shotgun metagenomic sequencing data. **wgs.tot**: total reads in milk whole genome sequencing data. **shotgun.cmv.reads**: total number of milk shotgun DNA sequencing reads (from WGS and/or SMS) mapping to CMV genome. **shotgun.cmv.reads**: total number of milk DNA sequencing reads from WGS and/or SMS. **shotgun.cmv.prop**: proportion of all milk shotgun DNA sequencing reads (from WGS and/or SMS) mapping to CMV genome. **shotgun.cmv.status**: milk sample designation as CMV+ or CMV- from shotgun sequencing data (WGS and/or SMS). **qpcr.copies.ml**: estimated CMV copies per mL of milk from qPCR. **qpcr.cmv.status**: milk sample designation as CMV+ or CMV- from qPCR. **Center**: study site (0=MN, 1=OK). **Parity**: number of previous births. **matage**: maternal age. **mat_bmi**: maternal pre-pregnancy BMI. **delivery_cat**: delivery mode (0: vaginal, 1: cesarean). **mateduc_cat**: maternal education category (0=high school/GED/associate’s, 1=bachelor’s degree, 2=graduate degree). **income_cat**: household income category (0 = less than $30k, 1 = between $30k to $90k, 2 = greater than $90k). **wlz_0, wlz_1, wlz_6**: infant weight-for-length Z-score at birth, 1 month, or 6 months of age. **laz_0, laz_1, laz_6**: infant length-for-age Z-score at birth, 1 month, or 6 months of age. **waz_0, waz_1, waz_6**: infant weight-for-age Z-score at birth, 1 month, or 6 months of age. **gdm**: gestational diabetes status during the focal pregnancy. **mat.white**: maternal self-identify as white/European-American. **ebf6mo**: exclusive breastfeeding status at 6 months postpartum. **inf.white**: infant self-identify (by mother) as white/European-American. **ebf3mo**: exclusive breastfeeding status at 3 months postpartum. **avg_totalhei**: average total Healthy Eating Index score from surveys during pregnancy, 1 month postpartum, and 3 months postpartum. **compfoods6mo**: complementary solid foods introduced to infants diet at 6 months of age. **gbs**: maternal Group B streptococcus status at delivery. **fecal_1mo_site**: collection site of 1 month infant fecal sample (home vs. study visit). **fecal_6mo_site**: collection site of 6 month infant fecal sample (home vs. study visit).

**Supplementary Data 2.** Sensitivity of main results to the proportion of CMV-mapped reads in milk used as a threshold to call a sample as CMV+, or to using qPCR CMV calls rather than shotgun sequencing. **Milk shotgun sequencing data**: Five thresholds were tested: the 1%, 10%, 25%, and 50% quantiles of the distribution of CMV-mapped read proportion within milk samples with at least 1 CMV-mapped read from shotgun sequencing data; **qPCR**: Results when CMV status was determined by qPCR. **Shotgun + qPCR:** Results when samples were defined as CMV+ by either qPCR or shotgun sequencing. All statistical tests reported in this table are two-sided and unadjusted for multiple tests.

**Supplementary Data 3**. Associations between milk CMV status and maternal traits. **CMV+, CMV-**: For binary traits, the percentage of participants in the CMV+/CMV- category with the trait is listed, with the number of participants with the trait listed in parentheses. For continuous traits, the mean trait value is given with the 2.5% and 97.5% percentiles in parentheses. **CMV+ N, CMV- N**: The number of participants in each CMV status group for each trait (some traits had missing data so the numbers were not the same for every trait). **P-value**: the P-value of the test for difference in proportions (binary traits, two-sided Fisher’s exact test) or difference in mean trait value (continuous traits, two-sided t-test) between CMV+ and CMV- groups. **Q-value**: Benjamini-Hochberg corrected P-value. **Trait**: the tested trait.

**Supplementary Data 4**. Output of DESeq2 testing for differential gene expression between CMV+ and CMV- milk samples. **baseMean**: the average of the normalized count values. **log2FoldChange**: the fold change (log2 scale) in CMV+ compared to CMV- milk samples. **lfcSE**: standard error of the log2 fold change. **stat**: value of the test statistic for significance testing. **pvalue**: two-sided P-value of significance testing for a differential gene expression between CMV+ and CMV- milk samples. **padj**: Adjusted P-value after Benjamini-Hochberg correction. **gene_id**: Ensembl gene ID. **gene_name**: Gene symbol.

**Supplementary Data 5**. Output of pathway enrichment testing. **Term**: tested gene ontology. **Overlap**: number of overlapping genes, number of genes in pathway. **P.value**: Enrichment test P-value (two-sided). **Adjusted.P.value**: Benjamini-Hochberg corrected P-value. **Odds.Ratio**: odds ratio of overlap. **Genes**: overlapping genes.

**Supplementary Data 6**. Output of DESeq2 testing for correlation between gene expression and the proportion of CMV-mapped reads in milk samples. **baseMean**: the average of the normalized count values. **log2FoldChange**: the fold change (log2 scale) change in gene expression for each standard deviation change in CMV-mapped read proportion. **lfcSE**: standard error of the log2 fold change. **stat**: value of the test statistic for significance testing. **pvalue**: two-sided P-value of significance testing for a differential gene expression between CMV+ and CMV- milk samples. **padj**: Adjusted P-value after Benjamini-Hochberg correction. **gene_id**: Ensembl gene ID. **gene_name**: Gene symbol.

**Supplementary Data 7**. Results of differential abundance testing of metabolites in CMV+ vs. CMV- milk samples. **beta**: estimated change in metabolite abundance in CMV+ compared to CMV- milk samples. **se**: standard error of effect estimate. **p**: two-sided P-value of effect estimate. **q**: Benjamini-Hochberg corrected P-value. **metabolite**: tested metabolite.

**Supplementary Data 8**. Results of association testing between principal components of the 1 and 6 month infant fecal microbiome taxon abundances and milk CMV status. **beta**: estimated effect of CMV+ vs. CMV- milk on PC value. **se**: standard error of effect estimate. **p**: two-sided P-value of effect estimate. **q**: Benjamini-Hochberg corrected P-value. **PC**: principal component tested. **timepoint**: 1 or 6 month infant fecal samples.

**Supplementary Data 9.** Results of association testing between principal components of the 1 and 6 month infant fecal microbial pathway abundances and milk CMV status. **beta**: estimated effect of CMV+ vs. CMV- milk on PC value. **se**: standard error of effect estimate. **p**: two-sided P-value of effect estimate. **q**: Benjamini-Hochberg corrected P-value. **PC**: principal component tested. **timepoint**: 1 or 6 month infant fecal samples.

**Supplementary Data 10.** Results of association testing between milk CMV status and microbial species abundances in infant fecal samples at 1 or 6 months of age, or in a model including both timepoints. Effect estimates are from a regression models of normalized centered-log ratio of abundances (see Methods). All p-values from two-sided tests. **taxon:** microbial species; **neg1mo.ra**: median relative abundance in 1 month old infants fed CMV- milk; **pos1mo.ra**: median relative abundance in 1 month old infants fed CMV+ milk; **est.1mo**: estimated effect of CMV+ vs. CMV- milk for 1-month time point. **se.1mo**: standard error of effect estimate for 1-month time point. **p.1mo**: P-value of effect estimate for 1-month time point. **neg6mo.ra**: median relative abundance in 6 month old infants fed CMV- milk; **pos6mo.ra**: median relative abundance in 1 month old infants fed CMV+ milk; **est.6mo**: estimated effect of CMV+ vs. CMV- milk for 1-month time point. **se.6mo**: standard error of effect estimate for 1-month time point. **p.6mo**: P-value of effect estimate for 1-month time point. **est.both**: estimated effect of CMV+ vs. CMV- milk from model including both 1 and 6 month timepoints. **se.both**: standard error of effect estimate from model including both 1 and 6 month timepoints. **p.both**: P-value of effect estimate from model including both 1 and 6 month timepoints. **q.both**: Benjamini-Hochberg corrected P-value from model including both 1 and 6 month timepoints.

**Supplementary Data 11.** Correlations between CMV-associated infant microbiome taxa (or PC3) and 1 month infant WLZ, 1 month milk kynurenine, or the proportion of CMV-mapped reads in CMV+ milk samples. **beta**: estimated effect per s.d. increase in tested trait on the microbiome trait. **se**: standard error of effect estimate. **p**: two-sided P-value of effect estimate. **taxon**: infant microbial taxon tested (or 1 month PC3). **trait**: tested trait (1 month infant WLZ, 1 month milk kynurenine, or the proportion of CMV-mapped reads in CMV+ milk samples).

**Supplementary Data 12.** Output of DESeq2 testing for correlations between expression of CMV-associated genes in milk at 1-month and abundance of CMV-associated microbial species in infant feces, at 1 or 6 months of age. All p-values are two-sided. **gene_id**: Ensembl gene ID; **gene_name:** gene symbol;  **taxon:** microbial species; **baseMean**: the average of the normalized count values for this gene in milk; **lfc.1mo.noCMV**: log2 fold-change gene expression in milk per standard deviation increase in normalized taxon abundance at 1 month, with milk CMV status not included as a covariate; **p.1mo.noCMV**: p-value for lfc.1mo.noCMV; **q.1mo.noCMV**: q-value for lfc.1mo.noCMV; **lfc.1mo.wCMV**: log2 fold-change gene expression in milk per standard deviation increase in normalized taxon abundance at 1 month, with milk CMV status included as a covariate; **p.1mo.wCMV**: p-value for lfc.1mo.wCMV; **q.1mo.wCMV**: q-value for lfc.1mo.wCMV; **lfc.6mo.noCMV**: log2 fold-change gene expression in milk per standard deviation increase in normalized taxon abundance at 6 month, with milk CMV status not included as a covariate; **p.6mo.noCMV**: p-value for lfc.6mo.noCMV; **q.6mo.noCMV**: q-value for lfc.1mo.noCMV; **lfc.6mo.wCMV**: log2 fold-change gene expression in milk per standard deviation increase in normalized taxon abundance at 6 month, with milk CMV status included as a covariate; **p.6mo.wCMV**: p-value for lfc.6mo.wCMV; **q.6mo.wCMV**: q-value for lfc.1mo.wCMV.

**Supplementary Data 13**. Results of association testing between milk CMV status and infant growth metrics at birth, 1 month, or 6 months of age. **est**: estimated effect of CMV+ vs. CMV- milk on infant trait. **se**: standard error of effect estimate. **p**: two-sided P-value of effect estimate. **trait:** infant growth metric and timepoint in the format trait_months; e.g. wlz_0 = weight-for-length at birth.

***Filename:*** CMV_milkGenomics_SupplementaryData14.zip

***Description:***

**Supplementary Data 14**

**milk_GeneExpr_TPM.txt**: gene expression levels estimated from RNA-sequencing of human milk, summarized as transcript per million (TPM). Rows are genes, columns are milk samples.

**milk_GeneExpr_counts.txt**: gene expression levels estimated from RNA-sequencing of human milk, summarized as counts. Rows are genes, columns are milk samples.

**metabolite_data.txt**: metabolite abundances from human milk. Rows are milk samples, columns are metabolites.

**infFecalMicrobiome_1month_taxonCLR.txt**: infant fecal microbiome taxon abundances (centered log ratio transformed) from 1 month of age. Rows are taxa, columns are infant fecal samples.

**infFecalMicrobiome_6month_taxonCLR.txt**: infant fecal microbiome taxon abundances (centered log ratio transformed) from 6 months of age. Rows are taxa, columns are infant fecal samples.
