# Supplementary material for: Human cytomegalovirus in breast milk is associated with milk composition and the infant gut microbiome and growth
Source: Nat Commun. 2024 Jul 23;15:6216. doi: 10.1038/s41467-024-50282-4 (PMC11266569; doi:10.1038/s41467-024-50282-4)
Supplement: Supplementary file 6 — Reporting Summary [file 41467_2024_50282_MOESM6_ESM.pdf]

nature portfolio

Corresponding author(s): Kelsey E. Johnson

Last updated by author(s): May 20, 2024

Reporting Summary

Nature Portfolio wishes to improve the reproducibility of the work that we publish. This form provides structure for consistency and transparency in reporting. For further information on Nature Portfolio policies, see our Editorial Policies and the Editorial Policy Checklist.

Statistics

For all statistical analyses, confirm that the following items are present in the figure legend, table legend, main text, or Methods section.

n/a	Confirmed	
☐	☒	The exact sample size (*n*) for each experimental group/condition, given as a discrete number and unit of measurement
☐	☒	A statement on whether measurements were taken from distinct samples or whether the same sample was measured repeatedly
☐	☒	The statistical test(s) used AND whether they are one- or two-sided<br>*Only common tests should be described solely by name; describe more complex techniques in the Methods section.*
☐	☒	A description of all covariates tested
☐	☒	A description of any assumptions or corrections, such as tests of normality and adjustment for multiple comparisons
☐	☒	A full description of the statistical parameters including central tendency (e.g. means) or other basic estimates (e.g. regression coefficient) AND variation (e.g. standard deviation) or associated estimates of uncertainty (e.g. confidence intervals)
☐	☒	For null hypothesis testing, the test statistic (e.g. *F*, *t*, *r*) with confidence intervals, effect sizes, degrees of freedom and *P* value noted<br>*Give P values as exact values whenever suitable.*
☒	☐	For Bayesian analysis, information on the choice of priors and Markov chain Monte Carlo settings
☒	☐	For hierarchical and complex designs, identification of the appropriate level for tests and full reporting of outcomes
☐	☒	Estimates of effect sizes (e.g. Cohen's *d*, Pearson's *r*), indicating how they were calculated

Our web collection on statistics for biologists contains articles on many of the points above.

Software and code

Policy information about availability of computer code

Data collection	N/A
Data analysis	No custom algorithms or software were used. Analyses used: RNA-SeQC v2.3.4, Bowtie2 v 2.2.4, DESeq2 v1.30.0, R version 4.3.1, enrichR v3.2, ggplot2 v3.4.2, ggbeeswarm v0.7.2, tidyr v1.3.0 , dplyr v1.1.2, vegan v2.6-4, Shi7 v1.0.1, BURST v0.99.7, HUMAnN 3.0.0, lavaan v0.6-17. Code to reproduce figures is available at https://github.com/kelsj/CMV_milk_genomics.

For manuscripts utilizing custom algorithms or software that are central to the research but not yet described in published literature, software must be made available to editors and reviewers. We strongly encourage code deposition in a community repository (e.g. GitHub). See the Nature Portfolio guidelines for submitting code & software for further information.

Data

Policy information about availability of data

All manuscripts must include a data availability statement. This statement should provide the following information, where applicable:

- Accession codes, unique identifiers, or web links for publicly available datasets
- A description of any restrictions on data availability
- For clinical datasets or third party data, please ensure that the statement adheres to our policy

Milk metabolite abundances, gene expression matrices, and microbial abundance tables are available as extended data tables (see descriptions in supplementary material). Milk RNA sequencing data is available at dbGaP study accession phs003408.v1.p1, and metagenomic sequencing data SRA accession PRJNA1019702.

Research involving human participants, their data, or biological material

Policy information about studies with human participants or human data. See also policy information about sex, gender (identity/presentation), and sexual orientation and race, ethnicity and racism.

Reporting on sex and gender	All adult participants were female as this study included lactating individuals recruited prenatally while gestating their infants, and thus were of female sex. We did not have information on gender identity. Infant growth Z-scores were sex-specific and so infant sex was not included as an additional covariate.
Reporting on race, ethnicity, or other socially relevant groupings	We used self-reported race as a covariate in our study, as there are previously reported discrepancies in the rate of CMV by race/ethnicity in the United States.
Population characteristics	Infant and maternal age, household income, education levels, parity, and self-identified race were included in our study.
Recruitment	Participants were recruited through their Ob/Gyn clinic during routine prenatal care.
Ethics oversight	All participants provided written informed consent and the study protocols were approved by institutional review boards at the University of Minnesota, HealthPartners Institute for Education and Research, and the University of Oklahoma Health Sciences Center.

Note that full information on the approval of the study protocol must also be provided in the manuscript.

Field-specific reporting

Please select the one below that is the best fit for your research. If you are not sure, read the appropriate sections before making your selection.

☒ Life sciences ☐ Behavioural & social sciences ☐ Ecological, evolutionary & environmental sciences

For a reference copy of the document with all sections, see nature.com/documents/nr-reporting-summary-flat.pdf

Life sciences study design

All studies must disclose on these points even when the disclosure is negative.

Sample size	Samples and data were collected prior to this study, so no prospective sample size calculations were done.
Data exclusions	Subjects/samples were excluded if they had missing data for each analysis.
Replication	N/A
Randomization	N/A
Blinding	N/A

Reporting for specific materials, systems and methods

We require information from authors about some types of materials, experimental systems and methods used in many studies. Here, indicate whether each material, system or method listed is relevant to your study. If you are not sure if a list item applies to your research, read the appropriate section before selecting a response.

Materials & experimental systems

n/a	Involved in the study	
☒	☐	Antibodies
☒	☐	Eukaryotic cell lines
☒	☐	Palaeontology and archaeology
☒	☐	Animals and other organisms
☒	☐	Clinical data
☒	☐	Dual use research of concern
☒	☐	Plants

Methods

n/a	Involved in the study	
☒	☐	ChIP-seq
☒	☐	Flow cytometry
☒	☐	MRI-based neuroimaging
